# Draft Genome of the Rice Coral *Montipora capitata* Obtained from Linked-Read Sequencing

**DOI:** 10.1093/gbe/evz135

**Published:** 2019-06-27

**Authors:** Martin Helmkampf, M Renee Bellinger, Scott M Geib, Sheina B Sim, Misaki Takabayashi

**Affiliations:** 1 Tropical Conservation Biology and Environmental Science, University of Hawaíi at Hilo; 2 Daniel K. Inouye U.S. Pacific Basin Agricultural Research Center, United States Department of Agriculture, Hilo, Hawaíi; 3 Marine Science Department, University of Hawaíi at Hilo; 4 Okinawa Institute of Science and Technology, Onna, Okinawa, Japan

**Keywords:** coral, genome assembly, linked-read sequencing, *Montipora capitata*

## Abstract

The rice coral, *Montipora capitata*, is widely distributed throughout the Indo-Pacific and comprises one of the most important reef-building species in the Hawaiian Islands. Here, we describe a de novo assembly of its genome based on a linked-read sequencing approach developed by 10x Genomics. The final draft assembly consisted of 27,870 scaffolds with a N50 size of 186 kb and contained a fairly complete set (81%) of metazoan benchmarking (BUSCO) genes. Based on haploid assembly size (615 Mb) and read *k*-mer profiles, we estimated the genome size to fall between 600 and 700 Mb, although the high fraction of repetitive sequence introduced considerable uncertainty. Repeat analysis indicated that 42% of the assembly consisted of interspersed, mostly unclassified repeats, and almost 3% tandem repeats. We also identified 36,691 protein-coding genes with a median coding sequence length of 807 bp, together spanning 7% of the assembly. The high repeat content and heterozygosity of the genome proved a challenging scenario for assembly, requiring additional steps to merge haplotypes and resulting in a higher than expected fragmentation at the scaffold level. Despite these challenges, the assembly turned out to be comparable in most quality measures to that of other available coral genomes while being considerably more cost-effective, especially with respect to long-read sequencing methods. Provided high-molecular-weight DNA is available, linked-read technology may thus serve as a valuable alternative capable of providing quality genome assemblies of nonmodel organisms.

## Introduction

Scleractinian or stony corals form the structural and trophic basis of coral reefs, which comprise some of the most diverse and productive ecosystems in the oceans (Spalding et al. 2001). Although providing livelihoods, coastal protection, and cultural value to millions of people around the tropics and subtropics (Moberg and Folke 1999), coral reefs are facing rapid global decline due to warming sea waters, ocean acidification, and local stressors (Hoegh-Guldberg et al. 2007; Hughes et al. 2017). From a scientific perspective, corals are noteworthy for engaging in a complex web of symbioses (reviewed in Gates and Ainsworth [2011]), in particular with photosynthetic dinoflagellates in the family Symbiodinaceae (LaJeunesse et al. 2018). This much studied partnership, which is driven by the exchange of carbon-rich compounds and other nutrients (reviewed in Davy et al. [2012]), is responsible for the enormous ecological and evolutionary success of scleractinian corals since their radiation in the mid-Triassic (Stolarski et al. 2011).

**Fig. 1.**
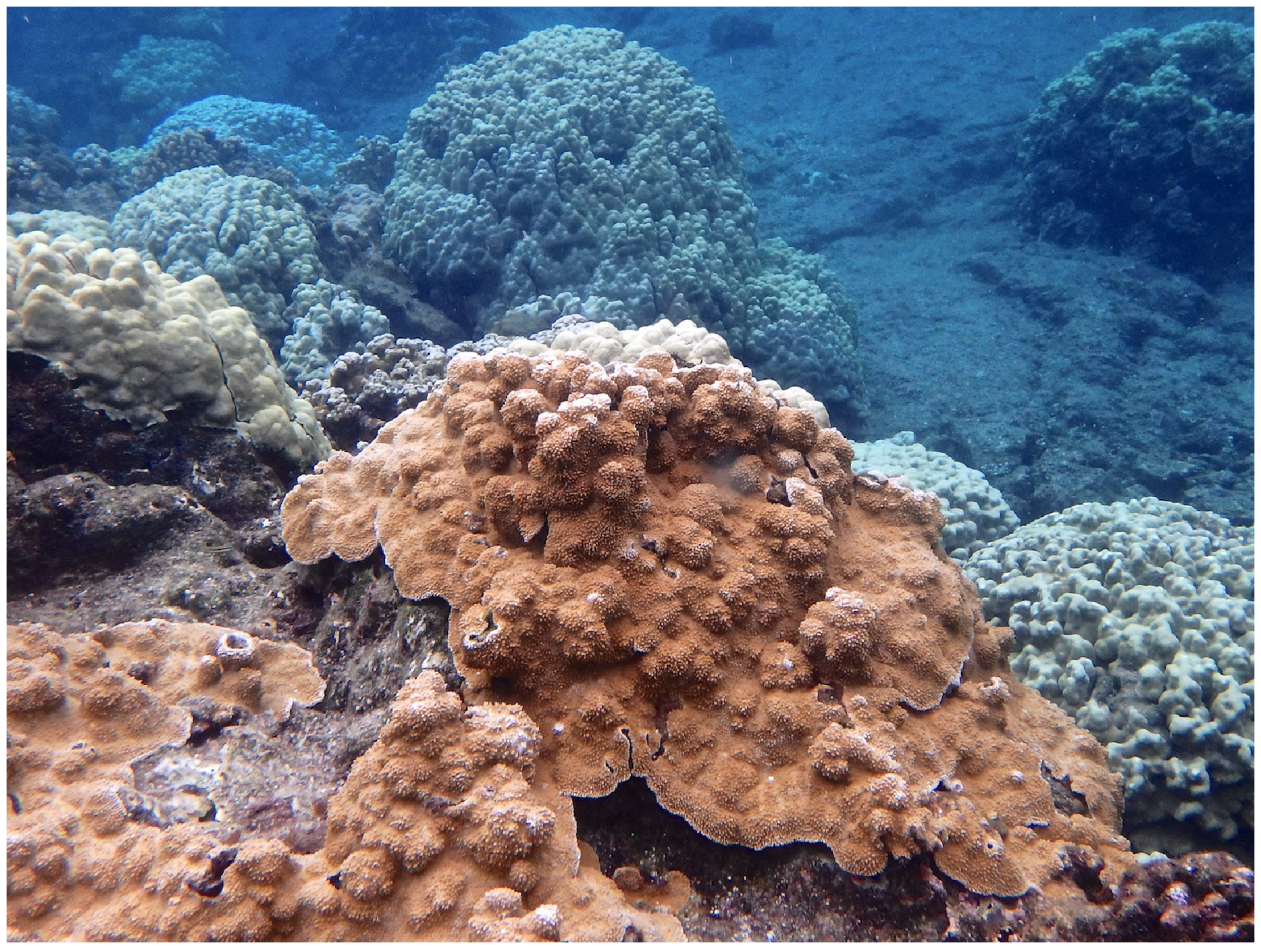
—Mature colony of the rice coral *Montipora capitata* (brown, foreground) at Wai‘ōpae tide pools, Eastern Hawai‘i Island. Photograph by Julia Stewart.

A better understanding of the evolutionary history of Scleractinia and the adaptations at the heart of their success requires deeper knowledge of the structure and function of their genomes (Bhattacharya et al. 2016). In addition, such information may also inform research of coral resilience and benefit efforts to mitigate the effects of the global coral health crisis. To contribute to these goals, we add a draft genome assembly of the rice coral *Montipora capitata* (fig. 1) to the growing body of genomic resources for reef-building corals (e.g., Shinzato et al. 2011; Voolstra et al. 2017). A widely distributed species in the family Acroporidae, *M. capitata* is native to the tropical north and central Pacific, as well as the Indian Ocean (DeVantier et al. 2018). It is especially common in the Hawaiian Islands, where it constitutes one of the major reef-builders, preferring turbulent, shallow waters down to a depth of 20 m. The species is characterized by minuscule corallites and small surface projections from which the common name rice coral is derived. Extreme phenotypic plasticity is also typical for the species, manifesting in a range of colony morphologies including encrusting, platelike, columnar, and branching forms. Like other corals worldwide, *M. capitata* is threatened by bleaching, coral diseases, and habitat degradation (DeVantier et al. 2018) and has been the focus of a number of studies concerning coral health, symbiont diversity, and other topics (e.g., Stat et al. 2011; Burns et al. 2013; Cunning et al. 2016; Frazier et al. 2017; Qiu et al. 2017). Recently, a first assembly of its genome obtained through a combination of Illumina and PacBio sequencing was published (Shumaker et al. 2019).

In this work, we explore the use of linked-read sequencing as a cost-effective alternative to conventional short- and long-read sequencing, with the goal to obtain a draft assembly of the *M. capitata* genome. This technology, developed by 10x Genomics (Pleasanton, CA), relies on partitioning long DNA molecules into emulsion droplets, and providing amplification products in each droplet with a shared barcode. Proceeding with standard library construction and Illumina sequencing, this creates groups of “linked reads” whose long DNA molecule of origin can be reconstructed, thereby improving assembly contiguity. Although the technology promises to combine the strengths of short-read sequencing (high per-base accuracy and throughput) and long-range information (identification of structural variants, haplotype phasing, and resolution of repetitive regions), it was primarily developed and assessed with model organisms, particularly human genomes (Marks et al. 2017; Weisenfeld et al. 2017). Testing it on a medium-sized, highly repetitive eukaryotic genome, we show that our results are comparable in most quality measures to conventional short-read sequencing, at a low cost, and discuss potential pitfalls and advantages of this approach. We describe the basic characteristics of the *M. capitata* genome, including its repertoire of repetitive DNA and protein-coding genes, and make the assembly available for public use to aid in future studies of coral evolution, ecology, and resilience.

## Materials and Methods

### Sample Collection

DNA was isolated from sperm of a single *M. capitata* colony collected at the former Wai‘ōpae tidepools, Hawai‘i Island (19°29′55″N, 154°49′06″W). Sampling activities were permitted by the Hawai‘i State Division of Aquatic Resources (Special Activity Permit 2016–33). Approximately ten colonies <20× 10 cm in size—young colonies encrusting smaller rocks and fragments that had broken off larger colonies—were identified and relocated to a sheltered, easily accessible tide pool on July 1, 2016. The date was chosen in advance of a mass spawning event expected around the new moon on July 4 (Kolinski and Cox 2003). At sunset of each subsequent day colonies were carefully transferred to individual plastic bins filled with 60 l of sea water, inspected in 30-min intervals, and returned to the tide pool at 22:00. During the peak of the spawning period on July 6 (light spawning was also observed the day before and after), gamete bundles floating at the surface in the bins were transferred to filtered sea water in 50-ml tubes using Pasteur pipettes. Samples were shaken lightly by hand to break up the bundles, transported to the lab on ice, and centrifuged gently to separate eggs and sperm. The bottom fraction containing the heavier sperm was transferred to 1.5-ml tubes by pipetting and stored as 500-µl aliquots at –20 °C.

### High-Molecular-Weight DNA Isolation and Sequencing

Genomic DNA was isolated from a sperm sample of a single colony by first embedding unlysed sperm in agarose, and then performing cell lysis and DNA purification in an agar plug, to maintain high-molecular-weight DNA. To do so, a 1.5% agarose solution was prepared in 0.5× TBE buffer (InCert Agarose, Lonza) by heating. Collected sperm were resuspended in nuclei isolation buffer (Cold Spring Harbor Protocols 2009, doi:10.1101/pdb.rec11656) and 50 µl of the resuspended sperm was mixed with 75 µl of the agarose solution. Before cooling, the sperm gel solution was cast into an agar plug mold (BioRad #1703713) and allowed to cool and solidify at 4 °C for 10 min. A series of gel plugs were prepared in this manner. Each cast gel plug was removed from the mold and placed in 2.5 ml of N-Laurylsarcosine Dodecyl Sulfate (NDS) buffer (Cold Spring Harbor Protocols 2009, doi:10.1101/pdb.rec11869; 0.01 M Tris-Cl at pH 9.5, 0.5 M EDTA), adding Proteinase K to a final concentration of 0.2 mg/mL, followed by overnight incubation at 50 °C in a water bath. The next morning, the NDS solution was decanted and plugs were washed once in 1× Tris–ethylenediaminetetraacetic acid (TE) buffer. To each plug, 3.0 ml of 1× TE buffer was added, and phenylmethylsulfonyl fluoride (PMSF; Millipore Sigma) in isopropanol was added to a final concentration of 0.1 mM, followed by gentle shaking for 1 h at room temperature to deactivate the Proteinase K. Fresh TE/PMSF solution was then added and the deactivation repeated for an additional hour. Next, four washes of 1× TE buffer without PMSF were performed, with gentle shaking for twenty minutes for each wash. The final extracted sperm samples in agar plugs were stored in 1× TE buffer until proceeding with library preparation and sequencing.

To isolate DNA, a single agar plug was treated with agarase. First the plug was equilibrated by washing twice with two volumes of 1× β-Agarase I Buffer (New England Biolabs) on ice for 30 min each. All buffer was removed from the tube, and the plug was gently melted by elevating the temperature on a thermal block. Four units of β-Agarase (New England Biolabs) were added, followed by incubation at 42 °C for several hours to dissolve the agarose. The resulting sample was viscous and stringy, indicative of high-molecular-weight DNA. The DNA sample was sent to the Genomic Services Lab at the HudsonAlpha Institute for Biotechnology, Huntsville. The quality of the DNA was determined via pulsed-field gel electrophoresis on a Pippin Pulse system using a 0.75% agarose gel and the 5-kb–430-kb protocol, and the quantity determined via Qubit. Approximately 1 ng of DNA was used as input for Chromium genome library preparation (v2 chemistry, 10x Genomics). The resulting library was indexed and sequenced on 0.5 lanes of a single flow cell on the Illumina HiSeq X Ten system, generating 150-bp paired-end reads.

### Genome Quality and Assembly

Illumina base call files were demultiplexed and converted to Fastq files by Long Ranger v2.1 mkfastq, a wrapper around Illumina’s bcl2fastq script provided by 10x Genomics. After removing internal barcodes with Long Ranger v2.1 basic, reads were evaluated with respect to quality and contamination based on GC content distribution using FastQC v0.11.4 by Babraham Bioinformatics. Overall genome characteristics, including genome size, heterozygosity, and repeat content were estimated from the distributions of *k*-mers in the debarcoded raw reads by Jellyfish v2.2.3 (Marçais and Kingsford 2011) and GenomeScope v1.0 (Vurture et al. 2017) with *k *=* *21 and a *k*-mer coverage cutoff of 10,000.

The genome assembly was constructed de novo by the 10x Genomics software package Supernova v2.1.1 (Weisenfeld et al. 2017) using all 357 million reads. We generated two additional assemblies based on 1) 291 million randomly selected reads to obtain 56× raw coverage as recommended by 10x Genomics and 2) 146 million randomly selected reads for half the recommended coverage. Only the initial assembly version was retained due to its superior quality, and two parallel fasta files representing the diploid assembly were generated with Supernova mkoutput in the pseudohap2 style based on contigs of 1,000 bp and greater. Each of these pseudohaplotype sets was composed of an arbitrary mix of maternal and paternal alleles. We chose the longer of the scaffold sets as a (mosaic) haploid representation of the genome for subsequent analyses. From this set we removed several artifacts: 1) stretches of Ns at the beginning and end of scaffolds, 2) scaffolds consisting entirely of Ns, 3) overlooked adaptor sequences (which were masked using a custom shell script), 4) duplicate scaffolds identified by megablast (applying a 98% identity cutoff over 95% query length; if unequal in length, shorter duplicates were discarded), and 5) scaffolds containing potential contaminants. We defined potentially contaminated scaffolds as those that produced BlastN hits against 2,787 RefSeq records of complete bacterial genomes available through NCBI’s FTP site on August 16, 2018, as well as two *Symbiodinium* sp. genome assemblies (*S. microadriaticum*, GCA_001939145.1, and *S.* sp. clade C, GCA_003297045.1), meeting the following cutoffs: *e*-value < 1 × 10^−10^, identity > 90%, length > 100 bp. As a final filtering step, the haploid assembly (with soft-masked repeats, see below for the repeat identification approach) was processed with the HaploMerger2 (v20161205, Huang et al. 2017) pipeline. This was done to further improve assembly quality by removing misjoins (steps A1–3), separating and merging alleles/connecting overlapping scaffolds (steps B1–5), and removing tandem errors (steps D1–3).

Basic statistics of the haplo-merged assembly and several other publicly available coral assemblies were calculated using Quast v4.6.1 (Gurevich et al. 2013), including: *Acropora digitifera* (Shinzato et al. 2011, GenBank assembly accession GCA_000222465.2), *M. capitata* (Shumaker et al. 2019), *Orbicella faveolata* (GCA_002042975.1), *Pocillopora damicornis* (GCA_003704095.1), *Porites rus* (GCA_900290455.1), and *Stylophora pistillata* (Voolstra et al. 2017, GCA_002571385.1). These assemblies were also assessed regarding gene space completeness using BUSCO v2.0.1 (Simão et al. 2015) and the Metazoa odb9 data set containing 978 genes. Finally, the *k*-mer spectra of the debarcoded raw reads were compared with those of the assembly using KAT v2.4.1 (Mapleson et al. 2016) in default Comp mode (*k = *27). The results were plotted for assembly validation and to investigate copy number variation between the assembly before and after haplo-merging.

### Genome Annotation

To analyze the repeat content of the *M. capitata* genome in detail, we generated a de novo repeat library from the Supernova assembly using RepeatModeler v1.0.11 (Smit and Hubley 2008–2015), which integrates RECON v1.08 (Bao and Eddy 2002) and RepeatScout v1.0.5 (Price et al. 2005) to find interspersed repeats (i.e., predominantly transposable elements). A second repeat library was constructed from the *M. capitata* assembly, the other coral assemblies listed above, and all ancestral eukaryotic repeats deposited in Repbase, but was discarded after this analysis proved to be less sensitive. Based on the *M. capitata* custom library, the *M. capitata* assembly was screened for repetitive elements using RepeatMasker v4.0.6 (Smit et al. 2013–2015) run with RMBlastN v2.2.27 before and after haplo-merging (for HaploMerger2 preprocessing and repeat annotation, respectively). Repeat models in the library as well as repetitive elements found in the assembly were classified according to Repbase (girinst.org, version 20150807) and CENSOR (Kohany et al. 2006). Tandem repeats were identified using the stand alone version of Tandem Repeats Finder v4.0.9 (Benson 1999) with the following settings: “Match = 2, Mismatching penalty = 7, Delta = 7, PM = 80, PI = 10, Minscore = 50, and MaxPeriod = 2,000.”

Protein-coding genes were predicted with the Augustus command line version 3.3.1 (Stanke et al. 2008). To create a training gene set, we mapped the *M. capitata* transcriptome identified by Frazier et al. 2017 (20,461 transcripts) to the genome assembly using BlastN. This data set only included transcripts with complete open reading frames and detectable transcription levels. Only transcripts that were at least 98% identical to the assembly over 300 bp were retained (because most alignments were interrupted by introns, the average identity and total length across all high-scoring segments per transcript-scaffold pair were considered). Transcripts with more than 80% identity to each other (TBlastX) were removed except one, as were those with matches in the custom repeat library (BlastN, *e*-value ≤ 1 × 10^−20^). The remaining set of 8,282 genes was further refined by PASA (Haas et al. 2013) implemented in web Augustus (Hoff and Stanke 2013), which resulted in a final training gene set of 1,584 genes. The Augustus meta parameters were then optimized for *M. capitata* based on this data set, reaching a sensitivity and specificity rate of 69% and 58% at the exon level, and 44% and 37% at the gene level, respectively. Alternative training sets we compiled, including various BUSCO-derived gene models, achieved lower sensitivity and specificity rates. For 1,383 (87%) training genes, we identified homologous proteins in the TrEMBL database using BlastP with *e*-value < 1 × 10^−5^ (50% covered at least 70% of the target length). Taking the taxonomic bias in the database and the frequency of taxonomically restricted genes into account, we consider this to validate most training genes as true protein-coding genes. We also created a hints file by 1) aligning the *M. capitata* transcriptome to the genome assembly using BLAT (Kent 2002) and 2) aligning 33,878 *A. digitifera* proteins (v1.1) to the assembly using Exonerate v2.2.0 (Slater and Birney 2005). Only the best hit per transcript or protein was retained, after applying a minimum coverage filter of 80% and 70%, respectively. This resulted in 12,169 transcript and 11,014 protein alignments. Guided by these as extrinsic evidence, we then predicted complete gene models in the assembly with soft-masked repeats (without low-complexity masking). Alternative transcripts and UTRs, which cannot be predicted as accurately as coding sequence, were not considered. Statistics for protein-coding genes for other coral species were taken from the NCBI Annotation Release 100, based on the NCBI Eukaryotic Genome Annotation Pipeline, or calculated from publicly available GFF files. Gene model completeness was assessed by BlastP searches of predicted proteins to *A. digitifera*, *O. faveolata*, *P. damicornis*, and *S. pistillata* proteins annotated by NCBI, and the *M. capitata* protein set by Shumaker et al. (2019) (*e*-value cutoff < 1 × 10^−5^).

## Results and Discussion

### Assembly and Genome Statistics

**Fig. 2.**
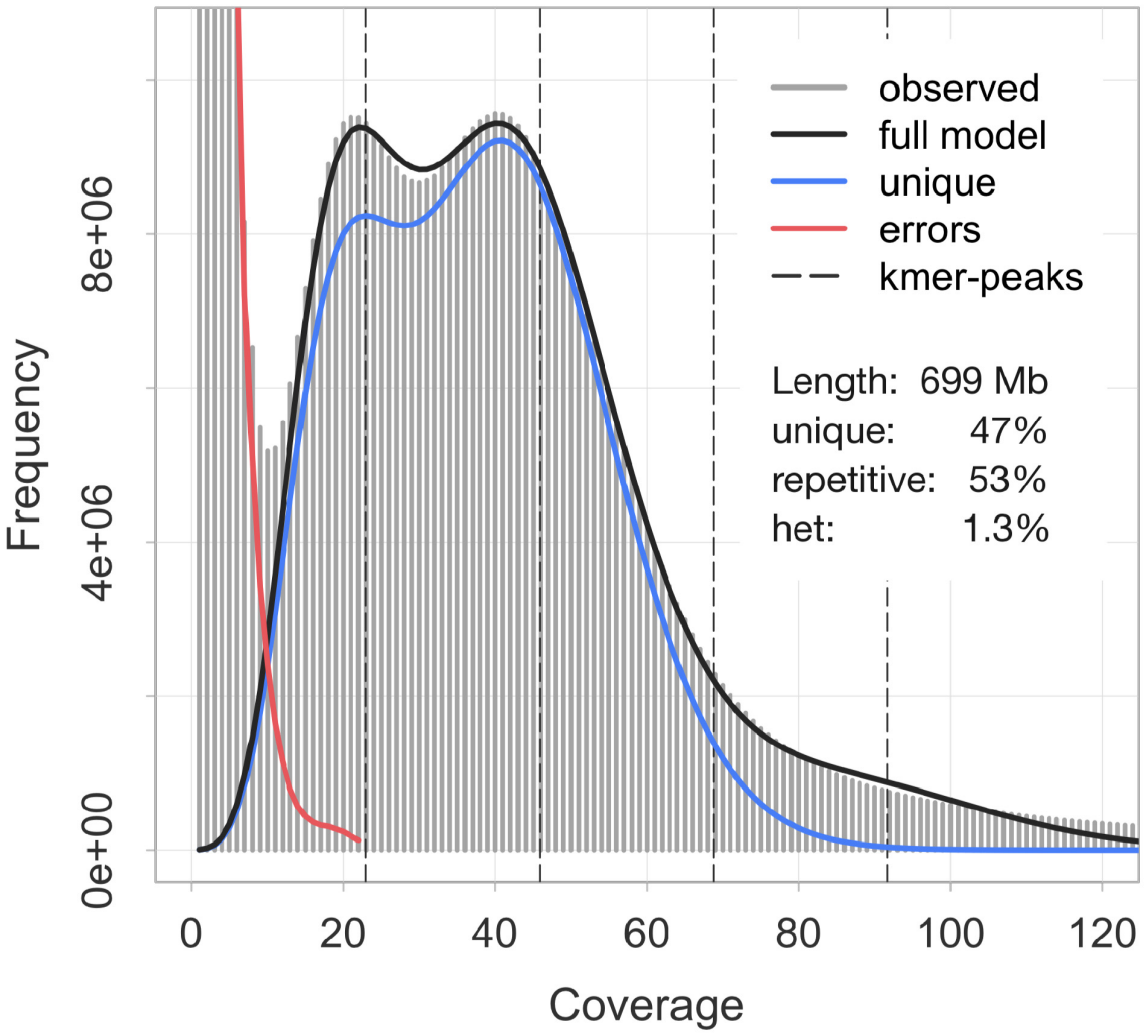
—*k*-mer profile (*k *=* *21) of the *Montipora capitata* genome raw reads as calculated by Jellyfish and GenomeScope. Light gray bars show the observed distribution, whereas the dark gray, blue, and red lines indicate the modeled distributions of *k*-mers representing the full genome, the unique fraction of the genome, and sequencing errors, respectively. Estimated genome characteristics include the genome size (length), nonrepetitive portion of the genome (unique), repetitive portion of the genome (repetitive), and genome heterozygosity (het).

We generated 357 million paired-end reads (2× 150 bp) from a linked-read library of a sperm sample of a single *M. capitata* specimen. The mean quality per read (Phred score) averaged 35.5. Based on the assembler’s genome size estimate, the effective coverage per base equaled 47× (counting both alleles; supplementary table 1, Supplementary Material online). According to the fraction of rare *k*-mers motifs in the reads (fig. 2), the sequencing error rate was 0.76%. We found no indication of systematic contamination with sequences from other organisms, as demonstrated by a single peak at 40% in the GC content distribution across all reads (supplementary fig. 1, Supplementary Material online). This result was corroborated at the assembly level, where sequences highly similar to bacterial or *Symbiodinium* genomes were almost entirely absent (see also below).

To build a de novo assembly using Supernova, we experimented with the number of input reads. Using all available reads (69× raw coverage) yielded slightly better results than limiting the raw coverage to 56× as recommended by 10x Genomics. In contrast, using only half the recommended reads resulted in significantly worse assembly metrics. We also found that the basic Supernova assembly contained a number of artifacts that had to be removed prior to subsequent analyses. Most notably, this included 6,506 typically short scaffolds (mean length = 3,177 bp) that were identical or nearly identical to other, usually longer scaffolds. The current version of the Supernova assembler thus did not seem to merge haplotypes completely. We also discarded a few scaffolds consisting entirely of unresolved sequence (Ns, *n* = 4), or containing short segments (100–400 bp) with high sequence similarity to bacterial or *Symbiodinium* genomes (*n* = 4). To investigate and further consolidate incompletely merged alleles, we processed the remaining 50,918 scaffolds (716 Mb) with HaploMerger2. The merged, final assembly comprised only 27,870 scaffolds (615 Mb), indicating the Supernova assembly harbored substantial potential to further merge haplotypes and connect overlapping scaffolds. As a result, the scaffold N50 size increased from 122 kb before haplo-merging to 186 kb, and the size of the largest scaffold from 1.99 to 2.05 Mb. Contigs were similarly affected (table 1). Because we wondered whether this approach may have been too aggressive, for example by collapsing similar sequences that are not alleles, we compared how many highly conserved metazoan genes could be recovered from the assembly using BUSCO. After haplo-merging, the number of single-copy benchmarking genes (*n* = 978) increased by 12 to 785, whereas the number of duplicated benchmarking genes decreased by 22 to 8. Although this equated a small net loss of ten genes (about 1%), it also confirmed that the haplo-merged assembly is a more accurate haploid representation of the genome. The effect was also evident in the *k*-mer spectra copy number plots of the assembly (supplementary fig. 2, Supplementary Material online). Homozygous content retained in duplicate by Supernova (purple, peak at 40×) was effectively removed by HaploMerger2, whereas the heterozygous fraction of the genome was further collapsed in the process (red and black, peak at 20×). In addition, the spectra demonstrate that rare *k*-mer motifs stemming from sequencing errors (black, low coverage content) were eliminated during assembly. The assembly also appeared free of sequence inconsistent with the reads, for example, due to phase switches or misjoined scaffolds.

**Table 1:** Basic Assembly Statistics for the *Montipora capitata* Genome, in Comparison to Other Coral Genome Assemblies (GenBank Accessions Are Given Where Available)

Organism	Assembly	Platform	Length (Mb)	Number	Largest (kb)	N50 Size (kb)	%GC	Ns
*M. capitata*	This study	Illumina (10×)	572	49,761	226	24.3	39.5	6,936
			615	27,870	2,051	185.5		
*M. capitata*	Shumaker et al. (2019)	PacBio + Illumina	—	—	—	—	39.6	—
			886	3,043	3,469	540.6		
*A. digitifera*	GCA_000222465.2	454 + Illumina	379	54,028	98	11.0	39.0	15,243
			447	2,420	2,550	483.6		
*O. faveolata*	GCA_002042975.1	Illumina	356	55,201	151	12.5	39.0	26,685
			486	1,932	4,772	1,162.4		
*P. damicornis*	GCA_003704095.1	Illumina	225	50,903	214	26.0	37.8	3,673
			234	4,392	2,168	326.1		
*Porites rus*	GCA_900290455.1	Unknown	332	81,420	66	5.3	38.9	29,322
			470	14,982	1,193	137.2		
*S. pistillata*	GCA_002571385.1	Illumina	358	37,778	250	20.5	38.5	10,536
			400	5,687	2,970	457.5		

Note.—Numbers on top refer to contigs, those below to scaffolds. “Ns” indicates the number of Ns per 100 kb. All statistics were computed directly from the assemblies rather than using published values to ensure comparability.

**Fig. 3.**
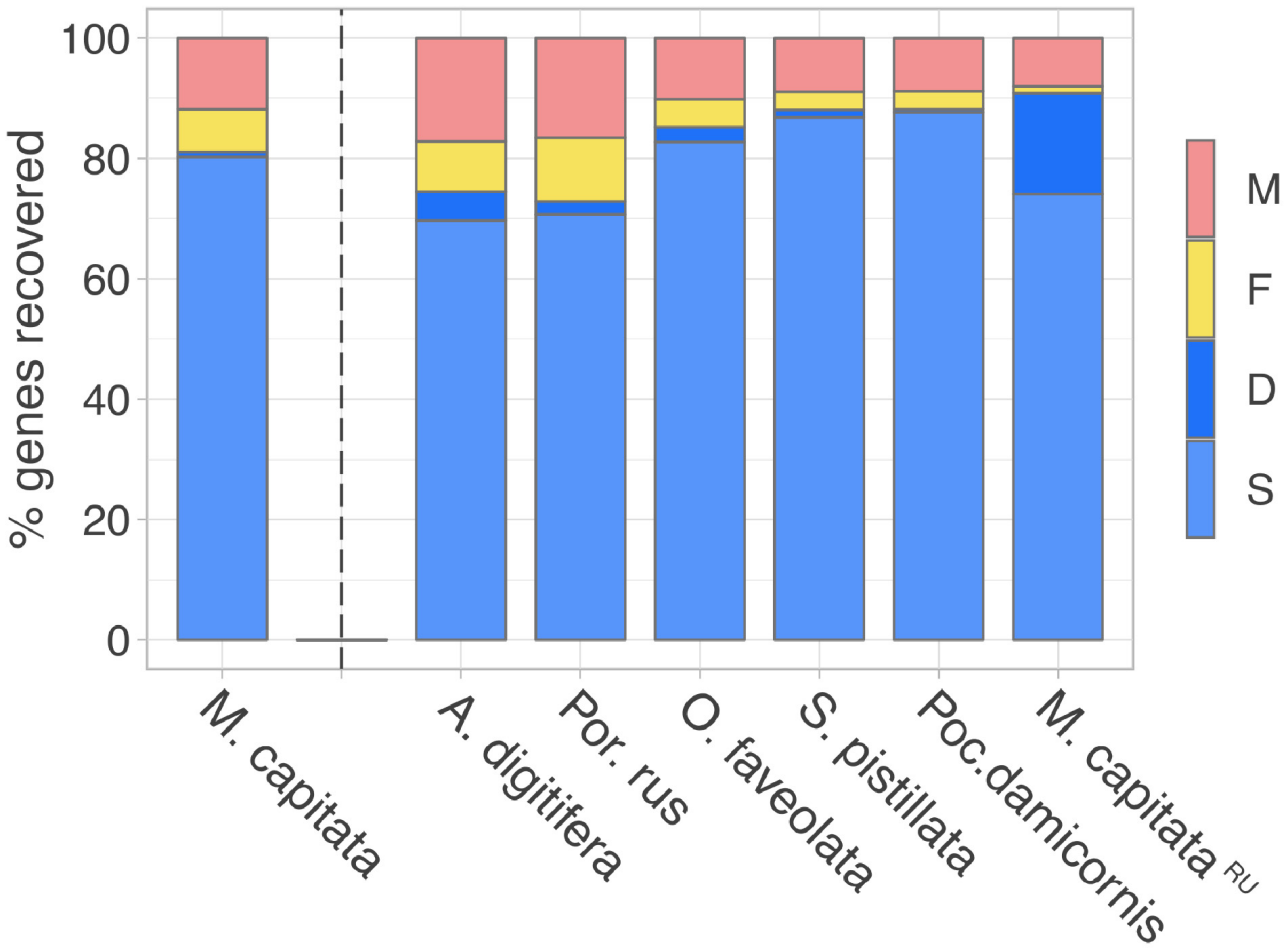
—Completeness of the *Montipora capitata* genome assembly (first column) assessed by the recovery of 978 metazoan benchmarking genes using BUSCO. For comparison, previously published coral genome assemblies were included in the analysis (see table 1, “^RU^” designates the *Montipora capitata* genome assembly by Shumaker et al. [2019]). Columns indicate the percentage of genes which were identified as single-copy (S), duplicated (D), fragmented (F), and missing (M) genes.

Regarding basic assembly metrics, the haplo-merged *M. capitata* assembly presented here compared well to the other sequenced coral genomes (table 1) at the contig level. Contig number and contig N50 size rated among the lowest and highest, respectively, of currently available coral genome assemblies. However, at the scaffold level, the assembly appears more fragmented. Although the largest scaffolds are within the same size range observed in other assemblies and the fraction of Ns is low, the assembly is split across a higher number of scaffolds (by a factor of ∼2–10), which is reflected in a comparatively lower scaffold N50 size. This circumstance may be due to differences in library types, sequencing technology, and assembly method. For instance, the other recently published *M. capitata* genome assembly (Shumaker et al. 2019) was obtained from a combination of PacBio and Illumina sequencing, a strategy that resulted in higher contiguity (table 1). However, differences in genome structure may also play a role in comparison to other species. In *M. capitata*, a high proportion of repeat sequence and considerable heterozygosity likely complicated the sequencing and assembly of the genome (see below). Despite this caveat, the assembly performed well in terms of recovering highly conserved metazoan genes using BUSCO (fig. 3). A total of 81% of the benchmarking genes could be retrieved completely, and another 7% at least partially. As mentioned above, the number of complete duplicate genes was extremely low, <1%. This result suggests that in terms of completeness, the *M. capitata* assembly is at least comparable to the first published coral genome, *A. digitifera* (Shinzato et al. 2011), for which we recovered 75% complete and 8% fragmented genes. Other coral genomes that were sequenced to greater depth more recently, including *S. pistillata* (Voolstra et al. 2017), achieved moderately higher benchmarks (>85% complete, 3–5% fragmented genes). The *M. capitata* assembly by Shumaker et al. (2019) also contained a higher number of benchmarking genes (91%), but a high fraction of those (17% in comparison to our <1%) were duplicated according to our BUSCO analyses. This may indicate that assembly—although representing a haploid genome as well—is less collapsed, retaining two alleles for a higher fraction of the genome.

We assessed basic attributes of the genome by analyzing the *k*-mer spectra of the reads (fig. 2, *k *=* *21), arriving at a genome size estimate for *M. capitata* of 699 Mb. According to the GenomeScope model, slightly more than half of this was classified as repetitive sequence. A distinct double peak in the distribution also indicated a relatively high rate of heterozygosity in the genome, 1.3%. Because we used a conservatively high *k*-mer coverage cutoff, the genome size estimate likely represents the upper bound, at least of genome content that is accessible by current sequencing techniques. Our assembly size is roughly consistent with this estimate, and decreased from 716 to 615 Mb after removing duplicate content that would otherwise result in overestimating haploid genome size. We thus conclude that the actual genome size of *M. capitata* is likely between 600 and 700 Mb. Although this is lower than previous genome size estimates for the species based on whole genome sequencing (886 Mb, Shumaker et al. 2019), accurate genome size estimates are notoriously difficult to achieve for highly repetitive and heterozygous diploid genomes. Whether the estimate by Shumaker et al. (2019) represents an overestimate (as suggested by the high percentage of duplicate BUSCO genes), or the size given here an underestimate (due to discarding high-coverage *k*-mer motifs or aggressive merging of repetitive sequence) remains an open question for now. Even the lower bound estimate suggests that *M. capitata* possesses a genome that is substantially larger than other available coral genomes. However, this still places it well within the range of 420–960 Mb that have been reported for Scleractinia using flow cytometry (Shinzato et al. 2011; Adachi et al. 2017).

### Annotated Genome Features

To better characterize the high fraction of repetitive DNA (fig. 2), we constructed custom repeat libraries from the *M. capitata* genome assembly and additional coral genomes. The *M. capitata*-specific library contained 1,894 repeat families, most of which were not archived in Repbase and thus remained uncharacterized. A total of 245 proved homologous to known non-LTR retrotransposons (predominantly LINEs, long interspersed nuclear elements), 89 to eukaryote DNA transposons, and 60 to LTR retrotransposons. Based on this library, we identified almost 1.03 million copies of transposable elements in the *M. capitata* genome, comprising about 257 Mb or 41.9% of the haplo-merged assembly (table 2). Most of these belonged to unclassified elements (193 Mb, or 31.4%). In addition to the RepeatMasker results, we detected more than 160,000 tandem repeats with a repeat unit size of up to 2,000 bp using Tandem Repeat Finder, spanning 16.5 Mb or 2.7% of the assembly. The unit size of these satellites averaged 26 bp, with a mean and maximum copy number of 9 and 386, respectively. Together, annotated interspersed and tandem repeats thus made up almost 45% of the assembly (table 2), just slightly short of the read *k*-mer based estimate (fig. 2). However, the relative contribution of repetitive DNA, and individual repeat classes to the genome, may be higher because heterochromatic regions are challenging to sequence and assemble reliably.

**Table 2:** Repetitive Elements Identified in the *Montipora capitata* Genome Assembly

	Number	Total Length (Mb)	Fraction of Assembly (%)
Tandem repeats	160,985	16.5	2.7
Interspersed repeats	1,028,006	257.4	41.9
DNA elements	31,667	11.6	1.9
LTR elements	14,753	10.0	1.6
Non-LTR elements	110,728	42.6	6.9
Unclassified	870,858	193.2	31.4

Gene prediction supported by transcription and homology-based evidence yielded 36,691 models of protein-coding genes (table 3), with a median length of 1,408 bp excluding UTRs. Typically, gene models were made up of two exons and one intron (median values, not counting UTRs), with a median length of 151 and 793 bp, respectively. Approximately 46% of gene models did not possess introns, and the median coding sequence per gene was 807 bp in length. These estimates are notably different from those reported for other coral species. The NCBI annotation pipeline arrived at a higher number of exons (4–5) and longer coding sequences (1,032–1,167 bp) for *A. digitifera*, *O. faveolata*, *P. damicornis*, and *S. pistillata* (table 3). Although not all of these variables are directly comparable because we did not annotate UTRs, we nonetheless wondered whether these differences were caused by artifacts in our assembly or annotation pipeline. However, our results were consistent with the median number of exons (2) and median coding region length (831 bp) published by Shumaker et al. (2019) for *M. capitata* using a different assembly and annotation approach. More than 91% of the predicted proteins had a BlastP match in *A. digitifera*, *O. faveolata*, *P. damicornis*, or *S. pistillata*. Approximately 54% covered at least 50% of the homologous target protein, and 27% covered 95% or more. When including *M. capitata* gene models provided by Shumaker et al. (2019), these ratios increased to 67% and 35%, respectively. Most gene models are therefore supported by homologous proteins identified in other coral genome assemblies. Finally, the total length of protein-coding sequence in the present genome (41 Mb) also provided evidence in favor of our findings because a similar range was observed in other corals (41–54 Mb, table 3). Assuming that a roughly similar spectrum of protein functions is needed across coral species, the extent of coding sequence identified in *M. capitata* would therefore be sufficient to fulfill these requirements. These lines of evidence suggest *M. capitata* genuinely differs from other sequenced corals with regard to typical gene structure, in particular by possessing fewer and shorter exons, and longer introns. Nonetheless, in other regards our results were in less explicable disagreement with previous studies. Notably, the number of genes we found (*n* = 36,691) differed markedly from both Shumaker et al.’s prediction for the species (*n* = 63,229), as well as four other coral species (19,935–26,073, table 3). Shumaker et al. (2019) showed that *M. capitata* possesses a large gene repertoire due to extensive gene family expansion in the lineage, while finding no evidence for whole genome duplication—another indication that the species’ genome evolved along a distinct trajectory, as is visible in its increased genome size and repeat content with regard to other corals. However, our results may indicate the gene inventory is not quite as large as reported by Shumaker et al., whose assembly possibly contains a higher fraction of diploid content, as discussed above. On the other hand, the discrepancy also highlights that gene prediction is challenging for a highly repetitive and heterozygous genome. Although we aimed to obtain as reliable predictions as possible by careful curation of the training gene set, leveraging extrinsic support, and masking repetitive sequence, we cannot exclude that the number of genes was biased by, for example, the splitting of gene models, which is more likely to occur in more fragmented assemblies. Indeed, we found a small number of very short, randomly selected gene models (<75 aa) to only partially match known metazoan genes (BlastP versus nr database), and to be located close to the edges of (usually very short) scaffolds. The BlastP results against other coral proteins reported above, while validating our annotations in general, also leave room for a moderate number of incomplete or split models to exist in the data set. We hope these can be addressed in future iterations of the annotation, which may profit from upgrades to the assembly, annotation methods, and manual gene model curation efforts.

**Table 3:** Protein-Coding Gene Features Annotated in the Genomes of *Montipora capitata* and Other Coral Species

	*M. capitata*	*M. capitata^RU^*	*A. digitifera*	*O. faveolata*	*P. damicornis*	*S. pistillata*
No. genes	36,691	63,227	26,060	25,916	19,935	24,833
Gene length	1,408	1,722	4,208	5,115	4,626	4,944
CDS length	807	831	1,032	1,068	1,173	1,167
Exon length	151	134	135	141	127	131
Intron length	793	879	585	583	473	572
No. exons/gene	2	2	4	4	5	5
CDS total (Mb)	40.5	73.5	36.4	39.6	32.7	41.0

Note.—Statistics were calculated from GFF files (present study and *M. capitata^RU^*, Shumaker et al. 2019) or taken from GenBank annotation reports (release 100, for corresponding assemblies see table 1). *Montipora capitata* gene models (first column) include coding exons only, so gene and exon length estimates are not directly comparable with annotations incorporating UTRs. Length estimates and number of exons per gene are median values.

### Utility and Performance of Linked-Read Sequencing

In this study, we present a nearly complete haploid genome assembly for the rice coral *M. capitata*. To our knowledge, this is one of the first published genomes of a nonmodel metazoan that was sequenced de novo using only 10x Genomics’ linked-read technology. With a contig N50 size and BUSCO scores (fig. 3) that are comparable to or exceed other coral genome assemblies (table 1), we demonstrate that this approach is capable of producing very useful results with untested organisms. Indeed, the method shares several advantages with conventional short-read sequencing on the Illumina platform, namely a lower per-base error rate and substantially lower cost than true long-read technology like Nanopore or PacBio’s single-molecule real-time sequencing (for this study, library construction plus sequencing were <1,600 USD in 2017). In contrast to adding long mate-pair libraries or using newer scaffolding techniques (e.g., Dovetail), library construction for 10x Genomics was straightforward. At the same time, linked reads promise to perform better at providing long-range information and resolving repetitive sequence than conventional short reads. However, our expectation of the high contiguity that can be achieved with linked-read sequencing was only partially met. At the scaffold level, the fragmentation of our assembly was relatively high, indicated by a scaffold N50 size that was two to three orders of magnitude lower than in other 10x Genomics studies (Weisenfeld et al. 2017; Hulse-Kemp et al. 2018), and also lower than what is typically achieved using short-read sequencing at high coverages coupled with mate-pair strategies to resolve repeats. We also observed a number of artifacts, namely the occurrence of (mostly short) scaffolds containing redundant sequence in the initial Supernova assembly. Even after removing these scaffolds, BUSCO duplication rates and *k*-mer spectra suggested that a significant fraction of duplicate heterozygous content remained in the assembly. We were able to remedy this by performing additional processing steps with HaploMerger2, a necessity that took away some of the convenience and ease-of-use offered by Supernova, and bears the risk of introducing additional artifacts. Two factors that are well known to negatively impact assembly contiguity and which have likely contributed to the artifacts we observed, are genome repetitiveness and heterozygosity (10x Genomics customer support, personal communication). Although genomes with high degrees of either have been assembled successfully (see e.g., Supernova 2.x support website), the *M. capitata* genome is to our knowledge the first that is both highly repetitive and heterozygous. In addition, Supernova has been optimized with the repeat landscape of human genomes in mind and may not perform well with the repeat size distribution found in the *M. capitata* genome (compare with Ott et al. [2018]). Although the software is designed to be heterozygosity-aware, it has mostly been applied to genomes with much lower heterozygosity (e.g., <0.1% in humans). In cases with higher heterozygosity (in our case, 1.3%) and in conjunction with a high repeat content, we showed that haplotypes may be merged only incompletely. A confounding factor may also have been the use of sperm as DNA source. Although collectively a pool of sperm cells should represent a single diploid genotype, each individual cell contains a different mosaic of maternal and paternal alleles after recombination during spermatogenesis. Frequent phase switches between contigs could pose a challenge to Supernova, which was designed to assemble somatic tissue DNA from a single diploid individual with low heterozygosity into long phaseblocks. Indeed, the phaseblock N50 size of our assembly was only slightly larger than the scaffold N50 size (see supplementary table 1, Supplementary Material online). Mosaicism, the existence of multiple genotypes in the same colony, has also been reported for several coral species (Schweinsberg et al. 2015). Unfortunately, the effects of recombination in germline samples and high rates of heterozygosity have not been explored yet with regard to 10x Genomics sequencing (customer support, personal communication). Future software updates may be better able to address the challenges of high heterozygosity and repetitiveness and to keep pace with the use of linked-read sequencing in a wider range of organisms and their diverse genome structures. Another important factor that likely affected assembly contiguity concerns the size distribution of the source DNA. Although experimental assays indicated sufficient quality with respect to the molecule length, it was calculated at only 25 kb based on the raw reads (supplementary table 1, Supplementary Material online). 10x Genomics recommends a mean molecule length of 50–100 kb, having demonstrated a strong effect on assembly quality (Zheng et al. 2016). Ensuring the use of high-molecular weight source DNA should therefore have priority when preparing samples for linked-read sequencing, and verifying the size distribution with several independent approaches may be advisable before library preparation.

Despite these challenges resulting in higher than expected assembly fragmentation, the overall quality proved sufficient for the analysis of basic features like the composition of the genome and its gene inventory. We are also confident that it will be suitable for future applications including the identification of genetic markers (e.g., microsatellites, SNPs), study of gene and gene family evolution, and more detailed comparative genomic analyses. Moreover, we expect that the possibility to identify structural variants and haplotype phases, though not taken advantage of here, will become standard in future genome projects. Linked-read sequencing therefore constitutes an effective, comparably easy and low-cost method for generating quality draft assemblies (Pajaanen et al. 2019), which might open the door for larger-scale (e.g., population) genomic projects when financial means and other resources are limited.

## Supplementary Material

Supplementary data are available at *Genome Biology and Evolution* online.

## Supplementary Material

evz135_Supplementary_Data

## Notes

**Data deposition:** This project has been deposited at GenBank under the accession PRJNA495325. The genome assembly is available under the accession RDEB00000000 (the version described in this paper is version RDEB01000000). Gene models have been deposited at the USDA Ag Data Commons, doi: 10.15482/USDA.ADC/1503958.
