# Draft Genome of the Rice CoralMontipora capitata Obtained from Linked-Read Sequencing

**DOI:** 10.1093/gbe/evz203

**Published:** 2019-11-04

**Authors:** Martin Helmkampf, M Renee Bellinger, Scott M Geib, Sheina B Sim, Misaki Takabayashi

*Genome Biol. Evol.* 11(7):2045–2054; doi:10.1093/gbe/evz135

An erroneous error was introduced in Table 1, row 4, all numbers should be positive instead of negative. This error has been corrected and the updated table appears online now.

The publisher regrets this error.

