# Supplementary material for: Draft Genome of the Rice Coral Montipora capitata Obtained from Linked-Read Sequencing
Source: Genome Biol Evol. 2019 Jun 27;11(7):2045–54. doi: 10.1093/gbe/evz135 (PMC6668484; doi:10.1093/gbe/evz135)

<u>Supplementary Material</u>

Draft genome of the rice coral *Montipora capitata* obtained from linked-read sequencing

Martin Helmkampf, M. Renee Bellinger, Scott M. Geib, Sheina B. Sim, Misaki Takabayashi

Content:

– Supplementary table 1: Supernova assembly report

– Supplementary fig. 1: Distribution of GC content across reads

– Supplementary fig. 2: Read *k*-mer spectra versus assembly copy number

Supplementary table 1 — Assembly report produced by Supernova v2.1.1

```
--------------------------------------------------------------------------------
SUMMARY
--------------------------------------------------------------------------------
- Mon Apr 01 05:48:08 2019
- [mcap10v4]
- software release = 2.1.1(6bb16452a)
- likely sequencers = HiSeq X
- assembly checksum = 8,889,190,959,852,679,109
--------------------------------------------------------------------------------
INPUT
-  356.99 M  = READS                 = number of reads; ideal 800M-1200M for human
-  139.50  b = MEAN READ LEN         = mean read length after trimming; ideal 140
-   69.12  x = RAW COV               = raw coverage; ideal ~56
-   47.05  x = EFFECTIVE COV         = effective read coverage; ideal ~42 for raw 56x
-   77.23  % = READ TWO Q30          = fraction of Q30 bases in read 2; ideal 75-85
-  320.00  b = MEDIAN INSERT         = median insert size; ideal 350-400
-   86.65  % = PROPER PAIRS          = fraction of proper read pairs; ideal >= 75
-    1.00    = BARCODE FRACTION      = fraction of barcodes used; between 0 and 1
-  779.92 Mb = EST GENOME SIZE       = estimated genome size
-   17.61  % = REPETITIVE FRAC       = genome repetitivity index
-    0.07  % = HIGH AT FRACTION      = high AT index
-   39.59  % = ASSEMBLY GC CONTENT   = GC content of assembly
-    0.11  % = DINUCLEOTIDE FRACTION = dinucleotide content
-   25.29 Kb = MOLECULE LEN          = weighted mean molecule size; ideal 50-100
-   73.29    = P10                   = molecule count extending 10 kb on both sides
-  275.00  b = HETDIST               = mean distance between heterozygous SNPs
-    4.98  % = UNBAR                 = fraction of reads that are not barcoded
-  292.00    = BARCODE N50           = N50 reads per barcode
-   20.83  % = DUPS                  = fraction of reads that are duplicates
-   48.08  % = PHASED                = nonduplicate and phased reads; ideal 45-50
--------------------------------------------------------------------------------
OUTPUT
-    9.76 K  = LONG SCAFFOLDS        = number of scaffolds >= 10 kb
-    9.36 Kb = EDGE N50              = N50 edge size
-   27.26 Kb = CONTIG N50            = N50 contig size
-  302.42 Kb = PHASEBLOCK N50        = N50 phase block size
-  179.37 Kb = SCAFFOLD N50          = N50 scaffold size
-   21.54  % = MISSING 10KB          = % of base assembly missing from scaffolds >= 10 kb
-  535.59 Mb = ASSEMBLY SIZE         = assembly size (only scaffolds >= 10 kb)
--------------------------------------------------------------------------------
ALARMS
- The length-weighted mean molecule length is 25291.83 bases. The molecule
length estimation was successful, however, ideally we would expect a larger
value. Standard methods starting from blood can yield 100 kb or larger DNA, but
it can be difficult to obtain long DNA from other sample types. Short molecules
may reduce the scaffold and phase block N50 length, and could result in
misassemblies. We have observed assembly quality to improve with longer DNA.
--------------------------------------------------------------------------------
```

Supplementary fig. 1 — Distribution of GC content across reads (FastQC, after barcode-trimming)

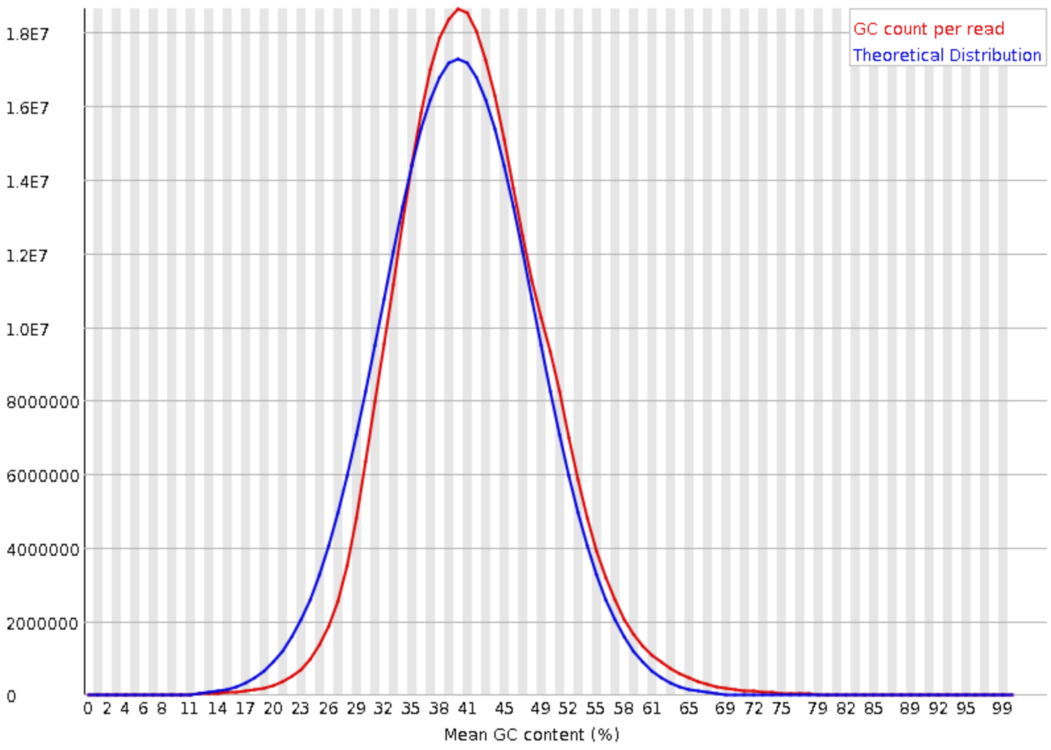

Supplementary fig. 2 — *k*-mer spectra of the raw reads obtained by KAT, with copy number in the assembly in color. (**A**) and (**B**) refer to the assembly before and after haplo-merging using HaploMerger2, respectively. Black bars indicate content that is not present in the assembly, red occurs once, and purple appears twice.

A

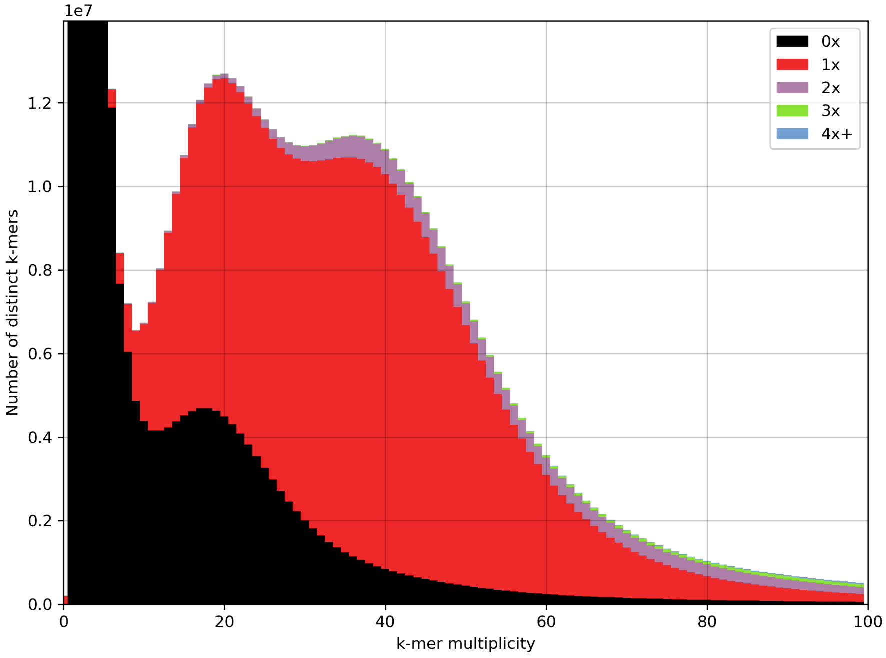

B

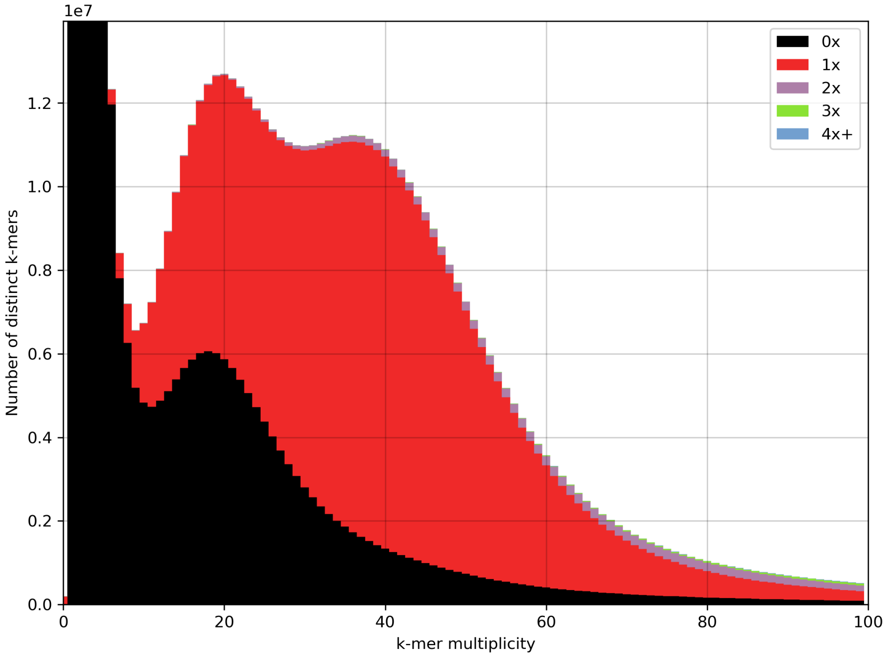

Supplement: evz135_Supplementary_Data [file evz135_supplementary_data.pdf]
